# Multi-site analysis of biosynthetic gene clusters from the periodontitis oral microbiome

**DOI:** 10.1099/jmm.0.001898

**Published:** 2024-10-08

**Authors:** Mohamad Koohi-Moghadam, Rory M. Watt, W. Keung Leung

**Affiliations:** 1 Division of Applied Oral Sciences & Community Dental Care, Faculty of Dentistry, The University of Hong Kong, Hong Kong SAR, PR China; 2 Department of Diagnostic Radiology, Li Ka Shing Faculty of Medicine, The University of Hong Kong, Pok Fu Lam, Hong Kong, PR China; 3 Division of Periodontology and Implant Dentistry, Faculty of Dentistry, The University of Hong Kong, Hong Kong SAR, PR China

**Keywords:** BGCs, metagenomic sequencing, microbial communities, oral microbiome, periodontitis, secondary metabolites

## Abstract

**Background.** Bacteria significantly influence human health and disease, with bacterial biosynthetic gene clusters (BGCs) being crucial in the microbiome–host and microbe–microbe interactions.

**Gap statement.** Despite extensive research into BGCs within the human gut microbiome, their roles in the oral microbiome are less understood.

**Aim.** This pilot study utilizes high-throughput shotgun metagenomic sequencing to examine the oral microbiota in different niches, particularly focusing on the association of BGCs with periodontitis.

**Methodology.** We analysed saliva, subgingival plaque and supragingival plaque samples from periodontitis patients (*n*=23) and controls (*n*=16). DNA was extracted from these samples using standardized protocols. The high-throughput shotgun metagenomic sequencing was then performed to obtain comprehensive genetic information from the microbial communities present in the samples.

**Results.** Our study identified 10 742 BGCs, with certain clusters being niche-specific. Notably, aryl polyenes and bacteriocins were the most prevalent BGCs identified. We discovered several ‘novel’ BGCs that are widely represented across various bacterial phyla and identified BGCs that had different distributions between periodontitis and control subjects. Our systematic approach unveiled the previously unexplored biosynthetic pathways that may be key players in periodontitis.

**Conclusions.** Our research expands the current metagenomic knowledge of the oral microbiota in both healthy and periodontally diseased states. These findings highlight the presence of novel biosynthetic pathways in the oral cavity and suggest a complex network of host–microbe and microbe–microbe interactions, potentially influencing periodontal disease. The BGCs identified in this study pave the way for future investigations into the role of small-molecule-mediated interactions within the human oral microbiota and their impact on periodontitis.

## Data Availability

All shotgun metagenome data described in this study were submitted to the Short Read Archive database (BioProject accession number: PRJNA932553). The sequence of all biosynthetic gene clusters and the results of antiSMASH outputs can be found at https://figshare.com/projects/Systematic_Analysis_of_Site-Specific_Biosynthetic_Gene_Clusters_BGCs_of_Oral_Microbiome_in_Periodontitis/157008.

## Introduction

Periodontitis is a prevalent and complex inflammatory disease that affects the supporting structures of the teeth, leading to progressive alveolar bone loss and, if left untreated, tooth loss [1]. The aetiology of periodontitis is multifactorial, with a significant contribution from the microbial communities present in the oral cavity. The human oral microbiome is a diverse consortium of bacteria, fungi, viruses and archaea that inhabit various niches within the oral cavity, including saliva and subgingival and supragingival plaque (Pl) [2,3]. These microbial communities are involved in both healthy and diseased states, influencing oral and systemic health through their metabolic activities and interactions with host tissues [4].

The functional potential of microbiomes can be explored through the lens of biosynthetic gene clusters (BGCs), which are groups of genes that collectively encode the production of secondary metabolites [5]. These metabolites have been implicated in a wide array of biological functions, such as antimicrobial defence, signalling and modulation of host immune responses [6]. In the oral cavity, BGC-derived metabolites may influence the onset and progression of periodontitis by shaping the composition and behaviour of the microbial community, as well as by directly interacting with the host’s immune system [7].

Despite the clinical importance of the oral microbiome in periodontitis, there has been a relative lack of studies focusing on BGCs within oral microbial communities compared to those in the gut. Advances in high-throughput sequencing technologies, particularly shotgun metagenomic sequencing, have begun to uncover the vast genetic potential encoded within the oral microbiome [7,8]. However, the specific roles that BGCs play in the context of oral health and periodontitis remain poorly characterized.

In this article, we present a systematic analysis of oral metagenome samples to survey BGCs within three different oral niches – saliva, subgingival Pl and supragingival Pl – from a cohort of 39 Chinese individuals, including 23 subjects with periodontitis and 16 controls. Despite the limited sample size, our focused approach allows for a meticulous examination of BGC profiles across different sites. Our results highlight the existence of both ubiquitous and niche-selective BGCs within the oral cavity and underscore the potential significance of specific BGCs in the pathogenesis of periodontitis.

## Method

### Subject recruitment

The inclusion criteria were as follows: (i) adults ≥18 years old, (ii) those in good general health and did not have any medical conditions that could potentially affect their oral microbiome and (iii) those without self-reported salivary gland disease or dry mouth of any type. Subjects were excluded if they (i) were smokers, (ii) were on medication, (ii) had a systemic disease, (iii) were pregnant or (iv) were undergoing orthodontic treatment.

### Clinical examination

Recruitment, consent and clinical examination were carried out at the Reception and Primary Care Clinic, Faculty of Dentistry, University of Hong Kong, between 1 October 2021 and 30 April 2022. All examinations were carried out by two calibrated examiners under the direction of W.K.L. Examinations were repeated in every tenth participant to assess inter-examiners’ reliability. Participants who provided written consent underwent a clinical examination, which was carried out by two calibrated examiners. Due to the coronavirus disease pandemic, all participants were prescribed a hydrogen peroxide-based pre-procedural mouth rinse on every attendance for cross-infection risk mitigation [9]. As described previously [10], the decayed, missing, and filled permanent teeth or surfaces index [11] was used to evaluate dental conditions, with decayed teeth defined at the International Caries Detection and Assessment System code 3 [localized enamel breakdown (without clinical visual signs of dentinal involvement)] or above [12,13].

We used a periodontal probe (PCP-UNC 15, Hu-Friedy Manufacturing Co., Chicago, IL, USA) to record the periodontal parameters from six sites of each tooth (mesio-buccal, mid-buccal, disto-buccal, mesio-lingual, mid-lingual and disto-lingual) excluding third molars. We checked whether supragingival Pl existed. The probing pocket depth (PPD) was determined by measuring the distance between the free gingival margin (FGM) and the base of the probing sulcus or pocket. Gingival recession (GR) was measured from the cementoenamel junction (CEJ) to the FGM and recorded as an integer: a positive value if the FGM was apical to the CEJ and a negative value if it was coronal to it. GR and PPD were added together to get the probing attachment level (PAL). Bleeding on probing (BOP) was marked as positive if bleeding started within 10–15 s after probing. Each patient’s BOP% (percentage of sites with BOP) and Pl% (percentage of sites with Pl) were calculated. The number of missing teeth (except for third molars) and presence and absence of furcation and mobility were also recorded [14]. The results from duplicate examinations on ten participants (every four from the third participant examined) showed that intra-examiner reliability on periodontal status (PPD/PAL in millimetres) was substantial (weighted kappa=0.618/0.810). Participants with any dental emergencies were immediately referred for proper management, whilst those with non-urgent oral problems were given referrals via the regular channels within the dental hospital.

### Sample collection

Subjects who qualified for the study were asked not to brush their teeth in the same morning when saliva/supra- or subgingival samples were taken, which was within 1–2 weeks after the recruitment, screening and examination. Participants were asked neither to eat nor to drink (except water) for at least 2 h before sample collection. First, 2 ml of the unstimulated saliva was collected according to Dawes [15]. For Pl collection, cotton rolls were used to isolate all teeth, which were air dried, and then, pooled supragingival Pl was collected from intact tooth crowns (excluding any decayed and restored tooth surfaces) using a sterile universal curette, followed by subgingival Pl sampling (pooled) using Gracey curettes from one deepest site on each quadrant.

In control participants, subgingival Pl was normally collected from a site with a PPD of 3/4 mm, and supragingival Pl was collected from a buccal site at a canine/premolar in each quadrant. In periodontitis participants, one deepest subgingival site per quadrant, i.e. PPD ≥5 mm, was sampled. In case the person had a quadrant without PPD ≥5 mm, an alternative non-adjacent deep pocket site (PPD ≥5 mm) in another quadrant was sampled as a replacement. Immediately after sample collection, all specimens were transferred to sterile 2-ml cryovials and quickly frozen for long-term preservation at −80 °C.

### DNA extraction

Frozen samples were thawed on ice. Using the QIAamp DNA Mini Kit (Qiagen), DNA was extracted according to the manufacturer’s instructions for Gram-positive bacteria. Forty-eight samples from 3 sites of the 39 subjects (16 healthy and 23 periodontitis) with purified DNA concentrations of more than 50 ng µl^−1^ have been sent for further shotgun sequencing. We excluded 41 samples from the analysis due to insufficient DNA concentrations.

### Next-generation sequencing

Samples that yielded sufficient amounts of DNA (≥50 ng µl^−1^) were selected to perform downstream shotgun metagenomic sequencing. The library preparation (paired-end sequencing of 151 bp) was performed at the Centre for PanorOmic Sciences, Genomics Core (The University of Hong Kong, LKS Faculty of Medicine) using the Illumina NovaSeq 6000 platform. Libraries were prepared based on the KAPA HyperPrep Kit (KR0961) protocol. Fifty nanograms of genomic DNA were fragmented using the Diagenode Bioruptor Pico system to a peak size of around 300 bp. End repair, A-tailing at the 3′ end and adaptor ligation with an integrated DNA technology dual-indexed unique molecular identifier adaptor system were performed on the fragmented DNAs. Dual solid-phase reversible immobilization was used to select an adapter-ligated library ranging in size from 300 to 750 bp. PCR was performed to enrich the libraries. For quality control analysis, Agilent Bioanalyzer, Qubit and qPCR were used to validate the enriched libraries. The libraries were denatured, and their concentration was optimized.

### BGC identification of oral bacteria

After generating the raw short reads, the KneadData pipeline (https://huttenhower.sph.harvard.edu/kneaddata/) was used to trim the barcodes, filter out low-quality reads and remove human reads from the samples. We used trimmomatic settings for a 4-base wide sliding window, with a minimum length of 90 bp and an average quality per base greater than 20. The final high-quality reads were assembled using MEGAHIT to generate the long contigs [16]. antiSMASH 6.0 was then used to identify BGCs within the long contigs [17]. antiSMASH detects BGCs consisting of multiple gene clusters of different types merged into a single large gene cluster. We developed a code to convert GenBank into FASTA using Biopython [18] python library to facilitate further bioinformatic analysis. Based on antiSMASH glossary [17], we mapped the discovered BGCs into six super-classes, namely, non-ribosomal peptides (NRPs), polyketides (PKs), ribosomally encoded and post-translationally modified peptides (RiPPs), saccharides, terpenes and others (Table S1, available in the online version of this article).

### Taxonomic assignment and phylogenetic tree construction

The taxonomic assignment of the assembly contigs was estimated using the Contig Annotation Tool (setting -sensitive -r 10 and -f 0.3) [19] based on the Genome Taxonomy Database [20]. Phylogenetic trees were constructed using PhyloT (https://phylot.biobyte.de), which were visualized using iTol (https://itol.embl.de/).

### BGC normalized abundance calculation

Bowtie2 [21] was used to quantify the number of hits to each BGC. We parsed the sequence alignment map output file from Bowtie2 to extract the number of hits. We considered hits with an alignment length of more than 60 bp and a sequence identity of more than 0.95. To compare the normalized abundance of each ${BGC}_{I}$ in control and periodontitis groups, we used the following equation:


$$Normalizedabundanceof{BGC}_{I}in{sample}_{j}=\frac{Totalnumberofhits{BGC}_{I}in{sample}_{j}}{Totalnumberofreadsin{sample}_{j}}\times 1,000,000\left(1\right)$$

### BGC novelty score calculation

To calculate BGC novelty, Biosynthetic Genes Super-Linear Clustering Engine (BiG-SLiCE) was executed in -query mode using a previously prepared dataset consisting of 1.2 million BGCs [22]. The resulting distance *d* represents the degree of similarity between a particular BGC and previously computed gene cluster families (GCFs), with a larger *d* suggesting greater uniqueness. We classified BGCs with a BiG-SLiCE score (*d*) greater than 1500 as novel BGCs. We used blast2GO [23] to perform gene ontology analysis of the novel unique clusters.

### BGC site specificity analysis

To facilitate comparisons, all sequences obtained for a specific domain were pooled and clustered according to sequence identity. We clustered the BGCs using CD-Hit (similarity >0.5) [24]. We classified the representative BGCs based on whether or not they were present in saliva, subgingival Pl, supragingival Pl or all three sites. For each oral site, the frequency of each of the representative BGCs was calculated. We used Venn diagrams to plot the results.

### BGC network analysis

To quantify the diversity of BGCs, we analysed all BGCs with Biosynthetic Gene Similarity Clustering and Prospecting Engine (BiG-SCAPE) [25], which groups similar clusters into a GCF. BiG-SCAPE generates a network based on the sequence similarity between the discovered BGCs and 1409 experimentally validated BGCs from the Minimum Information about a Biosynthetic Gene Cluster (MIBiG) repository [26]. We used domain sequence similarity score (weighted by sequence identity) to define a final distance metric for pairwise comparisons between BGCs. We saved the network in comma-separated values format and visualized with Cytoscape [27].

## Results

In this study, we conducted a comprehensive analysis of BGCs across three distinct oral niches, (1) unstimulated saliva, (2) supragingival Pl (multi-site, full-mouth) and (3) subgingival Pl (the deepest site per quadrant, pooled), from 39 ethnically Chinese individuals living in Hong Kong. This cohort included 23 patients diagnosed with periodontitis and 16 control subjects without severe periodontitis or advanced periodontal attachment loss [28]. We prioritized the analysis of 48 samples based on the criterion of having sufficient DNA concentrations (≥50 ng µl^−1^) for the purpose of shotgun metagenomic sequencing. The subsequent sequencing efforts produced a wealth of data that facilitated a deep exploration into the BGC composition and diversity within these oral microbial communities. The detailed characteristics and sequencing data of these samples are summarized in Tables 1 and S2, providing a foundation for the results we describe herein.

**Table 1.** Summary of 48 clinical samples analysed by shotgun metagenomic sequencing

Samples	Saliva	Subgingival Pl	Supragingival Pl	Excluded samples
Subjects				
Control subjects (16)	11	3	4	14*
Periodontitis subjects (23)	9	11	10	27*

*We excluded those samples with DNA concentrations less than 50 ng µl−1.

### Shotgun sequencing reveals diverse BGCs in the oral cavity

**Fig. 1.**
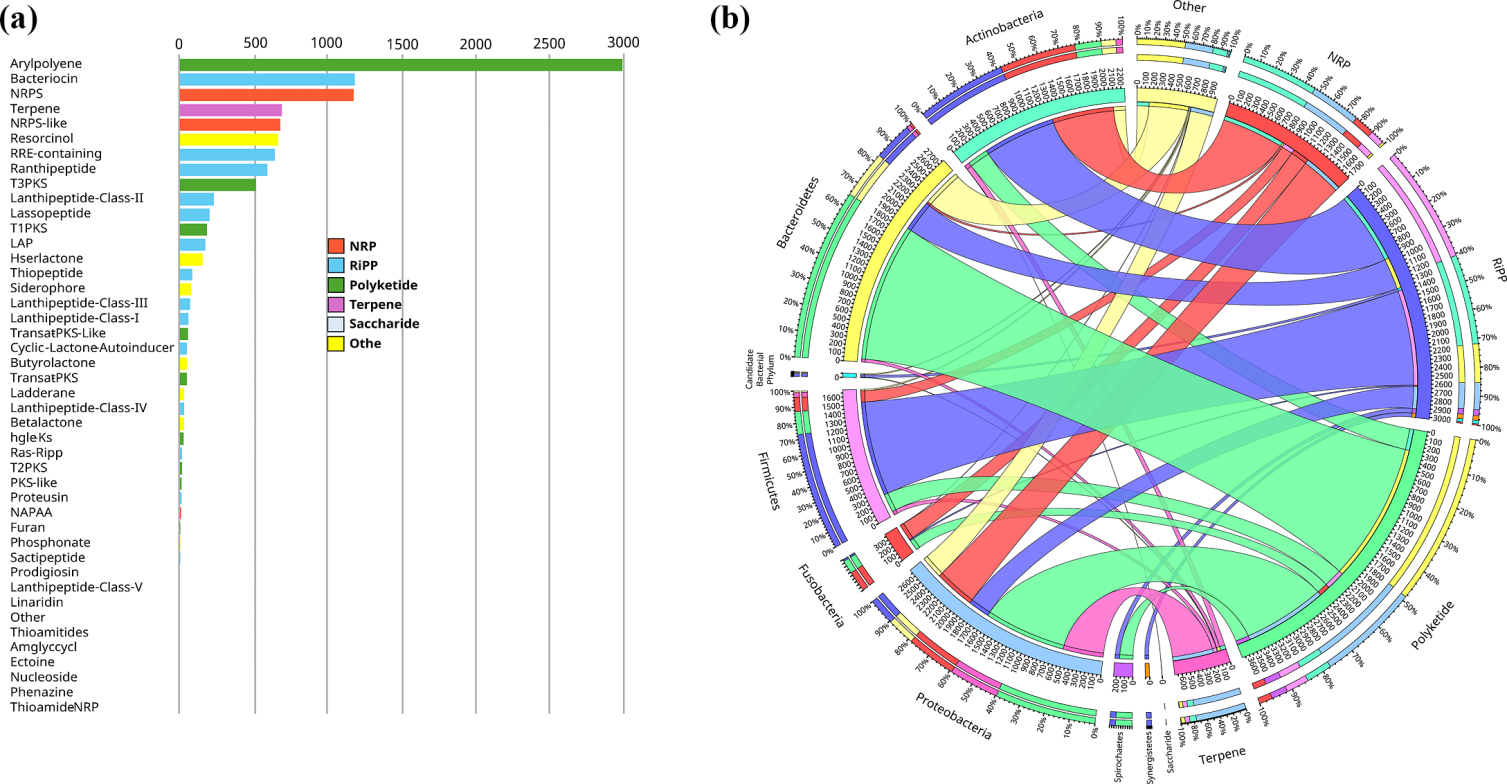
Distributions of BGCs identified within 48 pooled samples from periodontitis and healthy subjects. (**a**) The frequency and diversity of 44 BGC sub-types identified across the collected oral samples. (**b**) The distribution of BGCs across different bacterial phyla.

Shotgun sequencing of the 48 selected samples yielded an average of 94 076 902 ± 6 030 847 reads per sample, ranging from 79 059 540 to 108 878 790 (Table S3). Human reads were removed from each sample, and the remaining short reads were assembled to construct the bacterial genome contigs. These contigs were then analysed using antiSMASH 6.0 to identify BGCs [17]. A total of 10 742 BGCs were identified, representing 44 distinct groups (Table S4). These BGCs were categorized into six super-classes based on the antiSMASH glossary [17]: NRPs, PKs, RiPPs, saccharides, terpenes (isoprenoids) and ‘others’ (Fig. 1a).

PKs constituted the most abundant super-class, comprising 3833 BGCs (36% of all identified BGCs), with aryl polyenes (APEs) being the dominant subclass (2983 BGCs or 77% of all PKs). These were most prevalent in the bacterial phyla *Bacteroidetes*, *Proteobacteria*, *Actinobacteria*, *Firmicutes* and *Fusobacteria*, with the highest numbers found in Bacteroidetes (Fig. 1b). RiPPs formed the second most detected super-class with 3355 known BGCs (31% of all identified BGCs), including diverse sub-types such as bacteriocins, lantipeptides, ranthipeptides, sactipeptides and proteusins (Fig. 1a). Bacteriocins were the predominant RiPP sub-type, representing 1181 BGCs (35% of all RiPPs), primarily found within *Firmicutes* (Fig. 1b). The study also identified 1 866 NRP-class BGCs (17% of all identified BGCs), 692 terpene-class BGCs (7% of all identified BGCs) and 995 BGCs classified as ‘others’ (9% of all identified BGCs).

A detailed examination of BGC counts by type and phylum uncovered that three phyla, *Bacteroidetes* (*Bacteroidota*), *Proteobacteria* and *Actinobacteria* (*Actinomycetota*), were responsible for contributing over 70% of the identified BGCs. The *Firmicutes* and *Fusobacteria* phyla collectively accounted for approximately 20% of the total BGCs. The remainder of the BGCs were distributed among other phyla, which made up about 4% of the total, whilst 6% of the identified BGCs could not be classified at the phylum level (Fig. S1 A, B).

### Comparative analysis reveals distinct profiles of BGCs in oral bacteria from control and periodontitis patients

Investigating the variations in bacterial BGCs between healthy controls and periodontitis patients revealed distinct microbial metabolic potentials linked to the diseased state. The normalized abundance of BGCs was computed for each sample, and statistical comparisons were made using the Wilcoxon–Mann–Whitney test to determine the differences between the two groups. Our analysis identified several BGCs with significantly different abundances when comparing control individuals to those with periodontitis. These BGCs were associated with the synthesis of diverse small molecule types and originated from various bacterial taxa (refer to Fig. S2 for detailed taxonomy of BGCs).

**Fig. 2.**
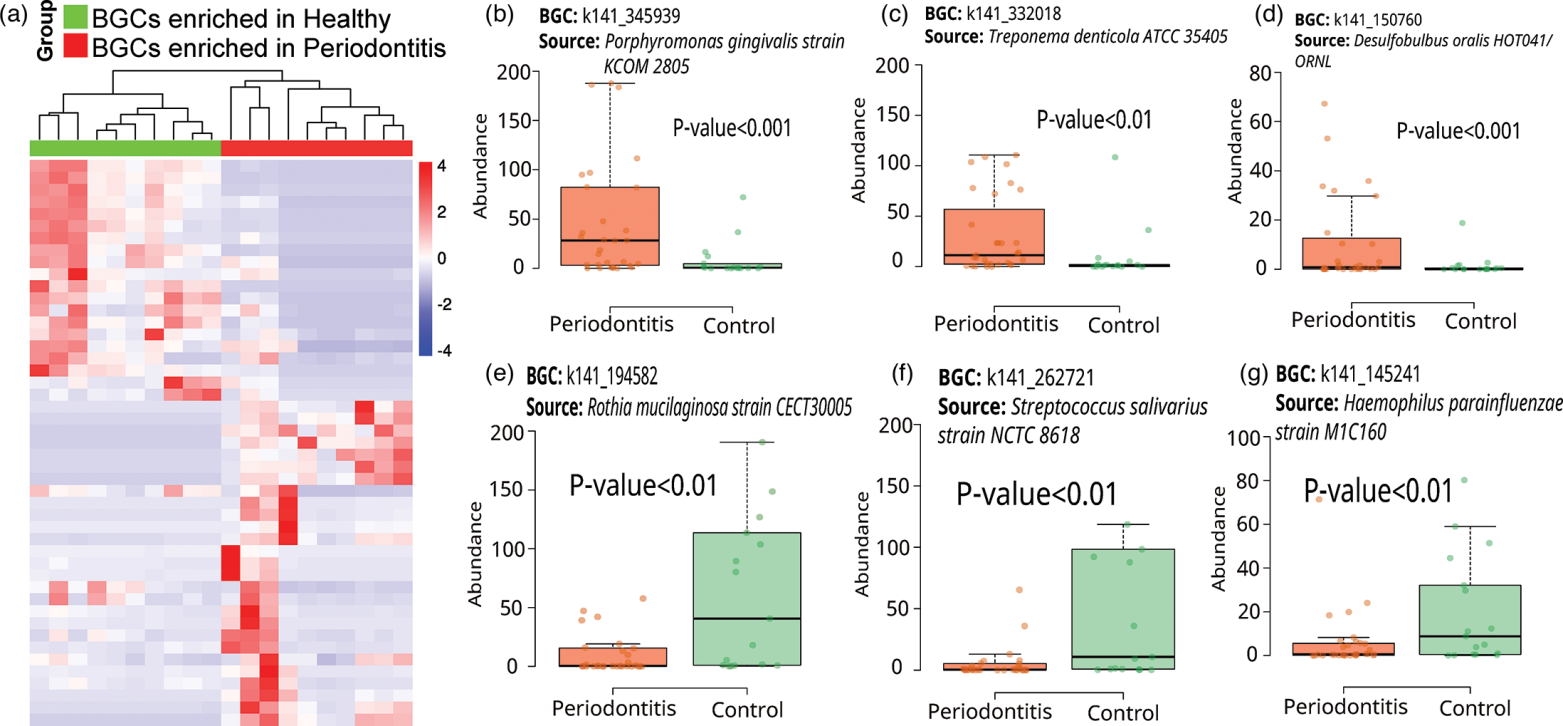
Comparative analysis of BGCs in oral samples from control and periodontitis subjects. (**a**) Heatmap depicting the differential distributions of the top 20 BGCs identified within control individuals and patients with periodontitis. The colour intensity represents the degree of enrichment in each group. (**b–d**) Depiction of three specific BGCs that show significant enrichment in periodontitis patients. (**e, f**) Illustration of three specific BGCs that are found to be enriched in the control group.

The top 20 BGCs, identified through a ranking based on their *P*-values, were visualized in a heatmap (Fig. 2a), offering a clear representation of their respective distributions between the control and periodontitis samples. Notably, certain BGCs, such as those responsible for the production of resorcinol, thiopeptide and NPRs, were predominantly found in pathogens like *Porphyromonas gingivalis*, *Treponema denticola* and *Desulfobulbus oralis*, which were more abundantly represented in periodontitis patients. Conversely, BGCs associated with the synthesis of NRPS synthetase, type III PK synthase (T3PKS) and betalactone were more prevalent in commensals like *Rothia mucilaginosa*, *Streptococcus salivarius* and *Haemophilus parainfluenzae*, which were frequently detected in the microbiomes of control subjects, as shown in Fig. 2b–g.

### Assessment of BGC diversity reveals high degree of novelty among different clusters

**Fig. 3.**
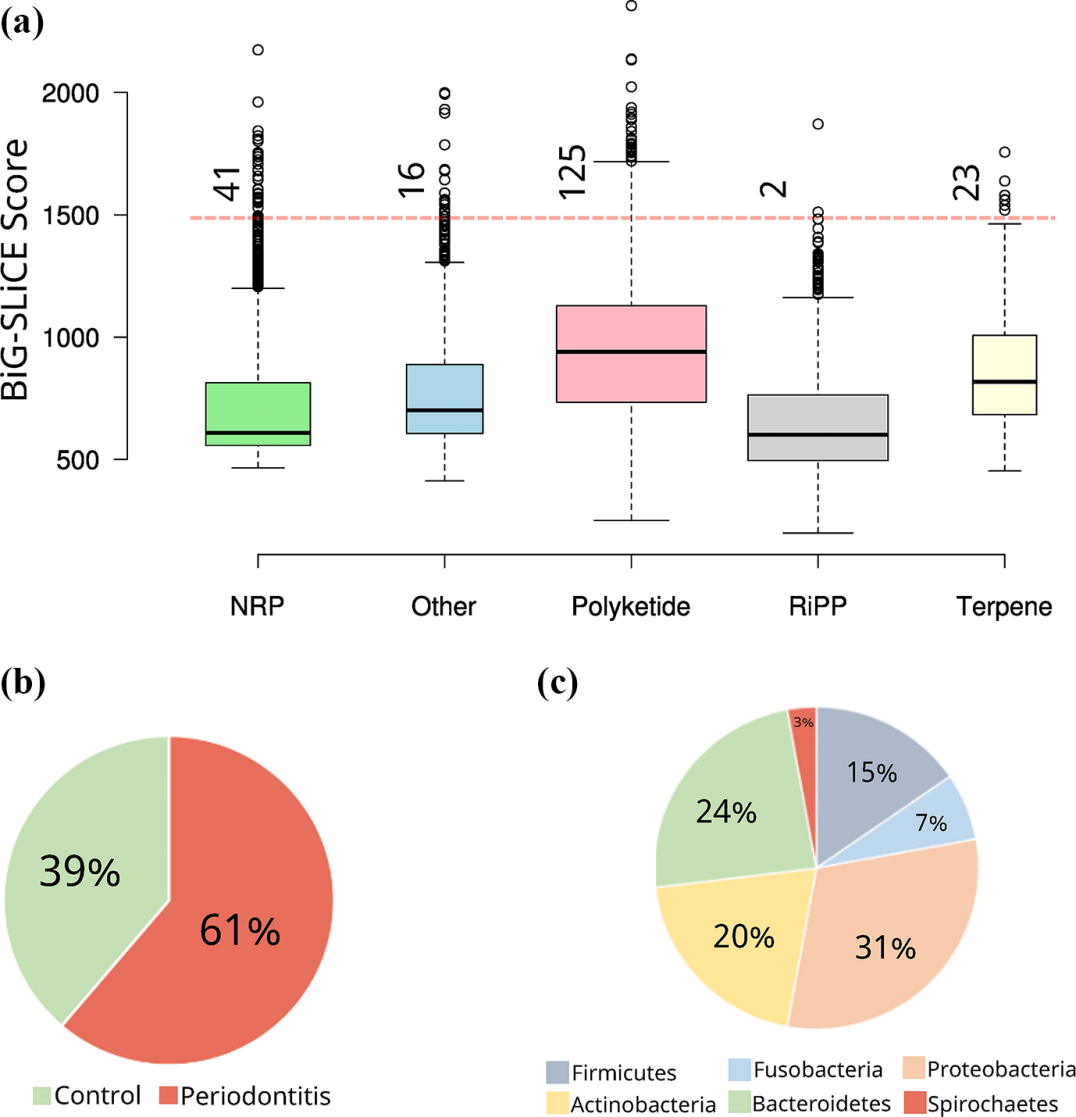
Characterization of novel BGCs in oral microbiomes from periodontitis and control subjects. (**a**) Boxplot representing the distribution of BiG-SLiCE distance scores for BGCs identified in the study. Scores are plotted for each BGC class, with a dashed red line indicating the novelty threshold at a distance score of greater than 1500, beyond which BGCs are considered highly divergent from known sequences. (**b**) Pie chart depicting the distribution of the 207 highly divergent BGCs between periodontitis and control samples. (**c**) Pie chart showing the distribution of the 207 novel BGCs across the 6 bacterial phyla identified as the primary genomic hosts for these divergent gene clusters.

In our investigation of BGC diversity, we utilized the computational tool BiG-SLiCE to systematically compare the distances of newly discovered BGC sequences against an extensive database of 1.2 million known BGCs [22]. The range of calculated distances (*d*) across BGC classes illustrates a spectrum of relatedness among the discovered BGCs. At one end of the spectrum are sequences that exhibit close genetic ties to known BGCs, indicating a high degree of similarity. At the other end of the spectrum are sequences with distant connections to any known BGCs, suggesting that they represent novel and potentially unique biosynthetic pathways (Fig. 3a).

We identified 207 BGCs with a distance greater than 1500, signalling an extreme divergence from the known collection of BGCs (as detailed in Table S5). This level of divergence suggests the existence of a substantial number of uncharacterized BGCs with the potential for producing novel bioactive compounds. The PK BGCs, known for their complex and pharmacologically important secondary metabolites, were particularly enriched for novelty, with 125 BGCs exceeding the threshold. This was followed by the NRP and terpene BGCs, which contributed 41 and 23 novel BGCs, respectively. We further studied the ecological context within which these novel BGCs were found. A significant majority, amounting to 61% of the novel BGCs, were detected within periodontitis-associated microbiome samples, whilst the remaining 39% were identified in control samples (as shown in Fig. 3b). Additionally, the bacterial phyla *Proteobacteria*, *Bacteroidetes* and *Actinobacteria* were the predominant hosts of these novel BGCs (Fig. 3c), indicating their significant contribution to BGC diversity.

We next checked the prevalence of these novel BGCs within each sample. Four BGCs were present in at least ten subjects, which suggests that they may play important roles in oral bacterial communities. BGCs k141_317058 and k141_328908 (encoded within *Neisseria* and *Ottowia*, respectively) were enriched in the control group, whilst k141_432967 and k141_157704 (encoded within *Desulfobulbus* and *Peptostreptococcus*, respectively) were enriched in the periodontitis group (Table S6). To have a deeper understanding of these BGCs products, we compared them with the known BGCs in the MIBiG databases.

BGC k141_317058 is similar to a BGC that produces biotin (vitamin B7 and vitamin H), an essential cofactor utilized by carboxylase enzymes that play key metabolic roles in microbes and humans [29]. BGC k141_328908 is predicted to synthesize polyhydroxyalkanoates (PHAs), biopolymers that are commonly produced by bacteria, which may be components of (extra-)cellular macrostructures or be used as a nutrient storage form. BGCs k141_432967 and k141_157704 are predicted to produce citrinin and phosphonoacetic acid, respectively. Citrinin is a PK mycotoxin (typically produced by fungi) that has been shown to have antimicrobial properties in addition to its cytotoxic effects [30].

### Comparative analysis demonstrates niche-specific distribution patterns of oral BGCs

**Fig. 4.**
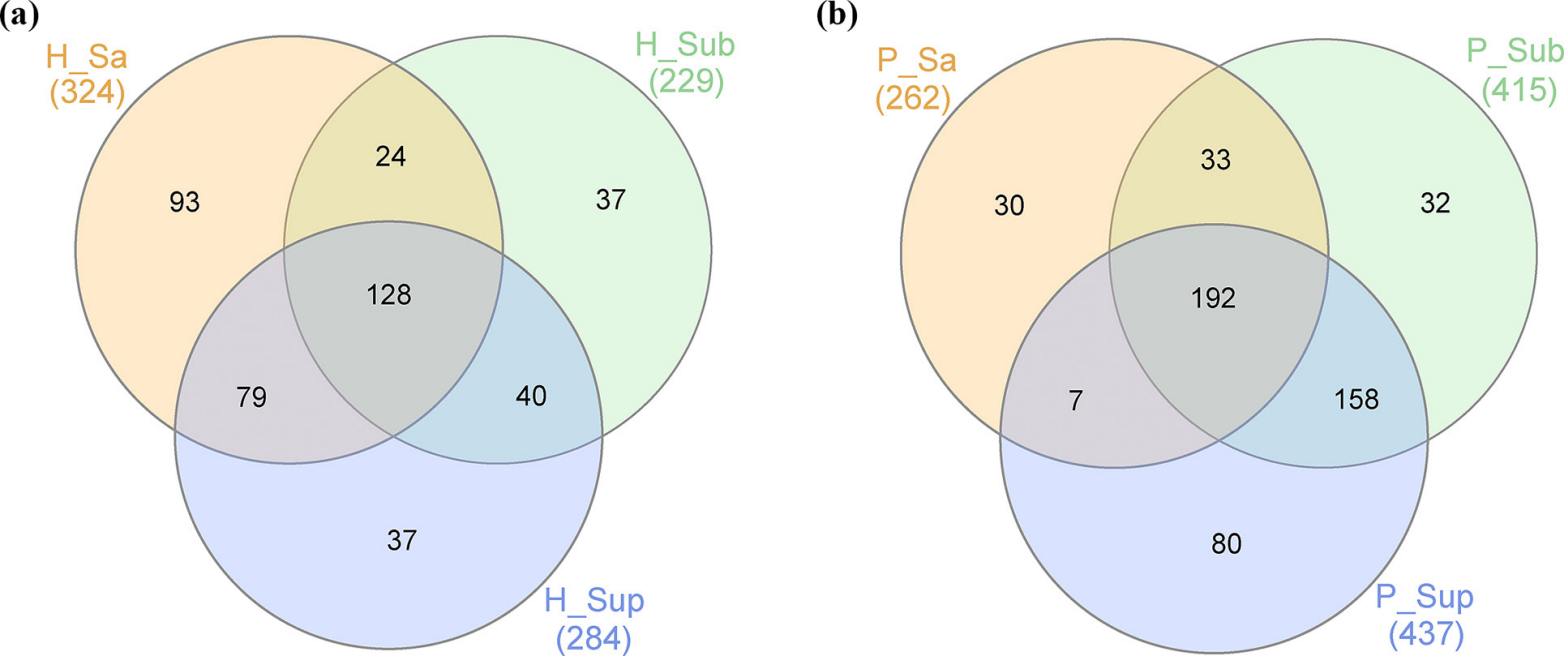
Site specificity of BGCs in the human oral microbiome in control and periodontitis subjects. Venn diagrams illustrating the distribution of BGCs across the six categorized groups representing distinct oral samples (C_Sa, control saliva sample; C_Sup, control supragingival Pl; C_Sub, control subgingival Pl; P_Sa, periodontitis saliva Pl; P_Sub, periodontitis subgingival Pl; P_Sup, periodontitis supragingival Pl).

By employing a comparative clustering approach, we categorized the BGCs based on their unique localization within saliva, subgingival Pl or supragingival Pl. The analysis involved pooling BGC sequences from various samples and clustering them according to sequence homology, as detailed in the Methods section. The patterns of distribution were visually represented using Venn diagrams to reveal the extent of BGC overlap across these distinct oral sites (Fig. 4).

Notably, a BGC identified as k141_164411, which is putatively responsible for producing a PK metabolite within the bacterium *Capnocytophaga gingivalis*, exhibited an exclusive presence in the supragingival and subgingival Pl from subjects with periodontitis. This particular BGC was detected in seven periodontitis cases, suggesting a potential role in the pathogenicity or the microbial ecology of periodontal disease niches. Conversely, another BGC, characterized by the cluster ID k141_302416 and presumably encoding an RiPP, was found solely within the *S. salivarius* contigs in saliva samples from healthy control individuals. This specificity indicates a unique adaptation of *S. salivarius* within the non-diseased oral environment, potentially contributing to the maintenance of oral health.

Some BGCs demonstrated a ubiquitous presence across all examined oral sites. One example is the BGC encoded by *Treponema medium* (BGC id: k141_23213), which was uniformly identified in saliva and supragingival and subgingival Pl samples from a cohort of 15 periodontitis patients. The prevalent distribution of this BGC across different oral niches in periodontitis patients provides a fascinating insight into the versatile capability of *T. medium* to establish itself within varied oral microenvironments.

### Network analysis identifies key bacterial gene clusters linked to periodontitis and control oral microbiomes

To uncover the potentially significant BGCs within the context of periodontitis and control oral microbiomes, we employed a robust network analysis. We identified the hub nodes – those with the highest degree of connectivity to facilitate the interpretation of these networks. Specifically, BGC K141_4883 emerged as a central hub within the control network, whilst K141_1504 and K141_316297 were identified as hubs in the periodontitis network (Fig. S3).

To deepen our understanding of these hub BGCs, we compared them with the known BGCs in the MIBiG database [26]. Specifically, the 61% similarity of BGC K141_4883 to a BGC in *S. salivarius* suggests conservation of biosynthetic pathways in this species, which is known for its prevalence in the healthy oral cavity. This could imply that the gene cluster plays a role in maintaining oral health or represents a common metabolic capability within this microbial community. Conversely, the 52% similarity of BGCs K141_1504 and K141_316297 to BGCs from *Centipeda periodontii* and *P. gingivalis*, respectively, organisms associated with periodontal disease, indicates that similar biosynthetic pathways may be active in the diseased state. These pathways could be related to the virulence, colonization or modification of the local environment to induce dysbiosis (Table 2).

**Table 2.** Comparison of the hub BGCs with known BGCs from the MIBiG database

Hub BGC	**Known BGC**	Similarity score	Type	Compound(s)	Host organism
K141_48883	BGC0000624.1	0.52	RiPP	Salivaricin CRL1328 α peptide, salivaricin CRL1328 β-peptide	*Lactobacillus salivarius*
k141_1504	BGC0001887.1	0.42	RiPP	Huazacin	*Bacillus thuringiensis* serovar *huazhongensis* BGSC 4BD1
k141_316297	BGC0000554.1	0.53	RiPP	SRO15-3108	*Streptomyces filamentosus* NRRL 15998

Further comparison of these hub nodes with BGCs with known secondary metabolite products enabled us to identify their putative products, adding another layer to our analysis. Our comparative results showed that the putative product of K141_4883 has similarity to the salivaricin CRL1328 β-peptide (score: 0.52), hinting at a role in bacteriocin production, which may contribute to microbial homeostasis in the oral cavity. K141_1504 was predicted to produce a compound similar to huazacin [31] (score: 0.42), a known antibacterial agent, whilst the putative product of K141_316297 was the molecule SRO15-3108 (score: 0.53), a lantipeptide class compound with reported antimicrobial activities [32].

## Discussion

### The role of BGCs in the oral microbiome and periodontitis

The findings of this study offer a novel perspective on the complexity and functional capabilities of the oral microbiome in both healthy and diseased states, particularly in the context of periodontitis. Our research has provided a comprehensive catalogue of BGCs across different oral niches, highlighting the intricate microbial metabolic landscape shaped by these gene clusters. APEs emerged as the most common family of BGCs in our samples. These compounds, present in several Gram-negative bacteria, have been implicated in resistance to oxidative stress and could induce biofilm formation through changes in cell envelope composition and regulatory cascades. The high concentration of APE BGCs in anaerobes populating the gingiva and tongue may reflect an adaptation to the highly reductive environments of these niches [33,34].

Bacteriocins, the second most abundant BGC type found in our study, are well known for their antimicrobial activities [35]. Their abundant presence in the oral cavity suggests that they could be significant players in microbial community dynamics, acting as mediators of competition between commensals and pathogens. The high transmissibility of bacteriocin BGCs may confer a certain degree of evolutionary advantage to their host species, contributing to rapid adaptations in the face of environmental pressures [36]. Furthermore, oral bacteria such as streptococci and lactobacilli, which produce bacteriocins, might play a role in suppressing pathogenic bacteria, thereby influencing oral and systemic health [37,38]. Moreover, the identification of novel BGCs, including those with a high degree of divergence from known BGCs, suggests that uncharacterized metabolic pathways may contribute to the pathogenesis of periodontitis. These novel clusters could represent a source of as-yet undiscovered bioactive compounds that influence the microbial ecology or the host response in periodontal disease. Beyond that, both APEs and bacteriocins also exhibit significant anti-fungal properties [34, 39]. This may possibly modulate oral *Candida* levels, which are known to have profound effects on oral ecosystems and host–microbe interactions [40].

### Disease association and niche adaptation of BGCs

Our analysis has identified BGCs that are differentially abundant between health and periodontitis, with potential implications for disease progression and the maintenance of oral health. The association of specific BGCs with known oral pathogens in periodontitis patients points to the possible roles in pathogenesis. The presence of BGCs from *P. gingivalis* in higher abundance in periodontitis samples aligns with the existing literature on periodontitis-associated pathogens [41]. The predicted synthesis of resorcinol by these BGCs and its potential involvement in biofilm formation could provide a mechanistic insight into how *P. gingivalis* establishes itself within the oral microbiome and contributes to disease progression [42]. Similarly, the increased abundance of BGCs producing thiopeptide and NRP-like compounds from *T. denticola* and *D. oralis*, respectively, in periodontitis samples, suggests that these compounds could influence the microbial community structure and biofilm dynamics [43]. On the other hand, the higher levels of BGCs encoding NRPS, T3PKS and betalactone in commensal bacteria within control samples indicate a potential protective role against periodontitis [44]. Bacteria such as *R. mucilaginosa* and *S. salivarius*, which are associated with anti-inflammatory properties, could contribute to a balanced immune response and homeostasis within the oral cavity [45,46].

The site specificity of certain BGCs, such as those uniquely found in *C. gingivalis* within subgingival Pl, suggests that some BGCs may confer niche-specific advantages or may be involved in the establishment and maintenance of pathogenic biofilms. Conversely, the specificity of a RiPP-encoding BGC within *S. salivarius* in saliva from healthy individuals could be indicative of a symbiotic relationship with the host, contributing to the stability of the oral microbiome. The observed overrepresentation of specific BGCs in periodontitis patients compared to controls is likely attributable to the distinct microbial communities present in these two groups. Whilst our study identifies BGCs with differential abundances in periodontitis patients, in future work, it will be crucial to determine whether these BGCs are actively transcribed during the disease process (discussed further below).

### Implications for microbial ecology and therapeutic development

Network analysis has revealed key bacterial gene clusters that are central within the BGC networks of periodontitis and control oral microbiomes. The identification of hub BGCs and their putative products provides valuable insights into the potential functional roles these clusters play within the oral microbiome. For instance, the similarity of a hub BGC in the control network to a known BGC from *S. salivarius* suggests a role in maintaining oral health, possibly through bacteriocin production. In contrast, hub BGCs associated with periodontitis may be involved in the disease process, offering potential targets for therapeutic intervention. For example, the predicted production of antibacterial compounds by some of these BGCs could be an adaptation mechanism by periodontal pathogens to outcompete commensal bacteria, facilitating dysbiosis and disease progression. We speculate that the increased abundance of BGCs predicted to encode antibacterial metabolites such as huazacin and the lantipeptide SRO15-3108 in the periodontitis group may be a result of a dysbiotic microbial community attempting to self-regulate or perhaps a consequence of the host inflammatory response (Table S6). It is conceivable that the inflammatory environment in periodontitis triggers an overproduction of these antimicrobial compounds, even in the presence of dysbiosis. In addition, the BGC that produces the mycotoxin citrinin may disrupt the balance of the oral microbiome by promoting dysbiosis and exacerbating periodontitis. Phosphonoacetic acid, on the other hand, is known for its antiviral properties. Its potential effects on the oral microbiome and periodontal health status are difficult to predict.

The two health-associated BGCs were predicted to synthesize biotin and PHAs, respectively. Supplementary biotin production may putatively improve the abilities of the host *Neisseria* bacterium to catabolize aas and synthesize important (fatty acid) metabolic intermediates, thus enhancing its survival abilities within competitive oral biofilm communities. In the context of oral health, PHAs could potentially contribute towards bacterial resilience or the structural integrity of biofilms that protect against pathogen colonization, thus helping maintain a healthy microbial balance in the oral cavity [47].

### Limitations and future directions

Whilst our study significantly advances our understanding of the biosynthetic potential of the oral microbiome, we acknowledge its limitations, including the small sample sizes and the potential underestimation of BGC diversity due to the resolution constraints of shotgun metagenomics. We must also point out that the overrepresentation of certain periodontitis-associated BGCs encoded by periodontopathogenic species may simply reflect the elevated levels of certain periodontopathogens present within diseased subjects (and vice versa). Thus, our study does not provide direct mechanistic evidence linking the products of specific BGCs (e.g. virulence factors) to periodontal disease pathologies and does not directly link the products of health-associated BGCs (e.g. bacteriocins and anti-inflammatory agents) with periodontal health. Our analysis at the bacterial genome level would benefit from complementary metatranscriptomic studies to characterize the niche-specific expression of these BGCs within the dynamic oral microbiomes associated with periodontal health or disease.

Looking ahead, there is a strong need to explore the therapeutic potential of novel BGCs discovered within the oral microbiome. The bioactive compounds these BGCs produce could lead to the development of innovative antimicrobial treatments or probiotic solutions for managing periodontal disease. For example, peptides from the oral microbiome, such as bacteriocins and lantibiotics [48], have shown significant antimicrobial properties, highlighting the potential of these bioactive compounds. These compounds have evolved to remain stable and active in the oral environment, making them particularly suitable for therapeutic applications. To that end, it will be necessary to expand our pilot research to include functional assays and validation studies. This will help translate our DNA-level insights into practical health solutions.

## Conclusions

This study has provided critical insight into the biosynthetic capabilities of the oral microbiome, particularly in relation to periodontal disease. We have catalogued a diverse range of BGCs, which are essential to the microbial dynamics of biofilm formation, competition and stress response. The presence of these BGCs in specific oral niches suggests their involvement in both the protection against and development of periodontitis, highlighting the dualistic nature of the oral microbiome in health and disease. However, it is important to underscore the limitations of our work. The small sample sizes used in this study may not capture the full diversity of the oral microbiome. This sample size limitation could also lead to a potential underrepresentation of the existing BGC diversity. Additionally, the resolution limits of shotgun metagenomics may have led to an underestimation of BGC diversity, potentially omitting less abundant or novel BGCs not previously characterized. Despite these challenges, our findings lay the groundwork for future investigations that can build on this knowledge, exploring the therapeutic potential of novel BGCs and integrating comprehensive ‘omics’ approaches, like metatranscriptomics and metabolomics, to fully elucidate the role of BGCs in periodontal health and disease.

## Supplementary material

10.1099/jmm.0.001898 Uncited Supplementary Material 1.

10.1099/jmm.0.001898 Uncited Table S1.

10.1099/jmm.0.001898 Uncited Table S2.

10.1099/jmm.0.001898 Uncited Table S3.

10.1099/jmm.0.001898 Uncited Table S4.

## Abbreviations

APEs: aryl polyenes
BGCs: biosynthetic gene clusters
BiG-SLiCE: Biosynthetic Genes Super-Linear Clustering Engine
BOP: bleeding on probing
CEJ: cementoenamel junction
FGM: free gingival margin
GCFs: gene cluster families
GR: gingival recession
MIBiG: Minimum Information about a Biosynthetic Gene Cluster
NRPs: non-ribosomal peptides
PAL: probing attachment level
PHAs: polyhydroxyalkanoates
PKs: polyketides
Pl: plaque
PPD: probing pocket depth
RiPPs: ribosomally encoded and post-translationally modified peptides
T3PKS: type III PK synthase
