# Supplementary material for: Multi-site analysis of biosynthetic gene clusters from the periodontitis oral microbiome
Source: J Med Microbiol. 2024 Oct 8;73(10):001898. doi: 10.1099/jmm.0.001898 (PMC12453396; doi:10.1099/jmm.0.001898)
Supplement: Uncited Supplementary Material 1. [file jmm-73-01898-s001.pdf]

Supplementary Figures:

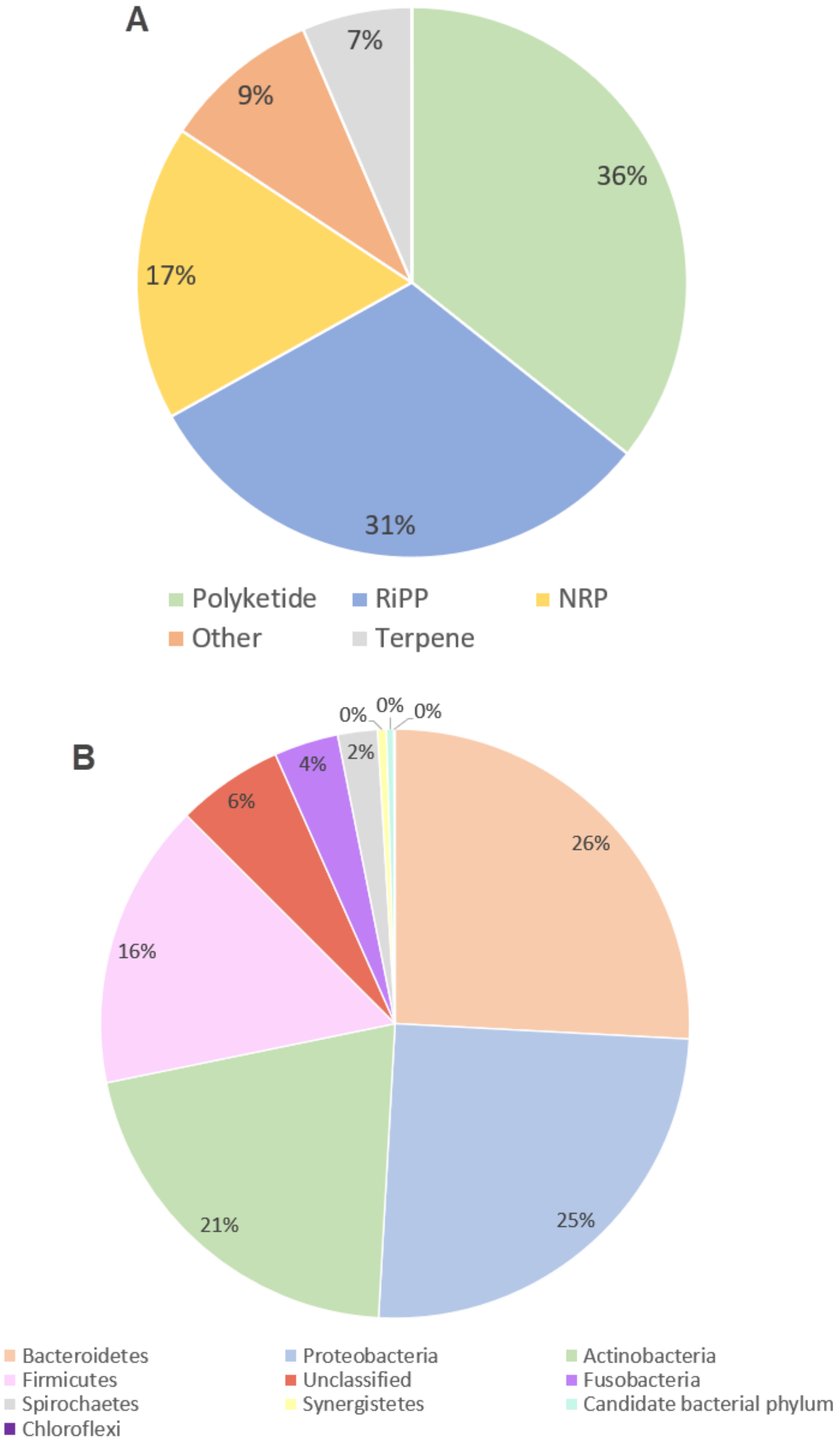

Figure S1. Profile of oral biosynthetic gene clusters (BGCs) in samples with purified DNA concentrations ≥ 50 ng/µL.

(A) Distributions of BGCs based on the type of product formed. (B) Distributions of BGCs based on the bacterial phylum they originate from.

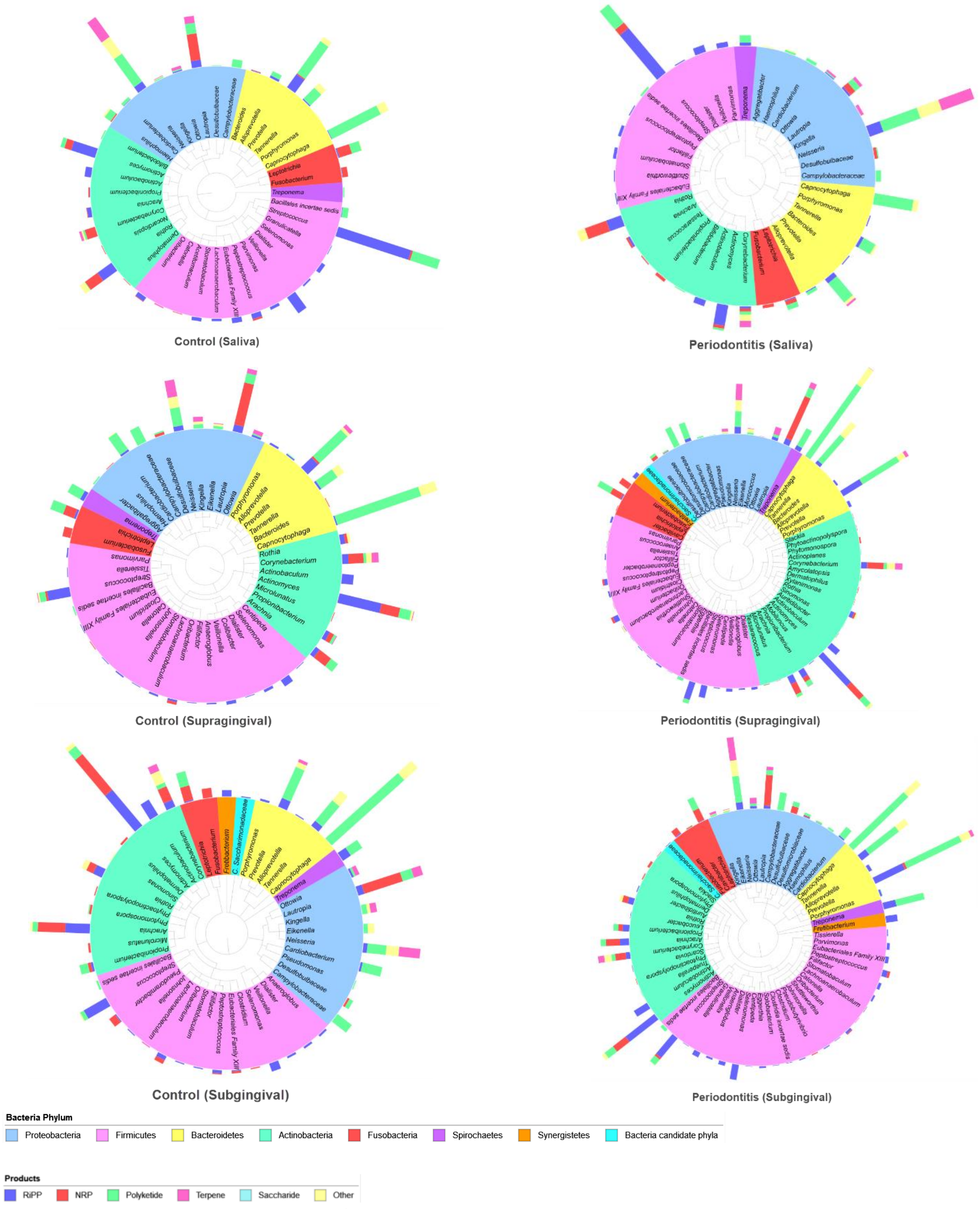

Figure S2. Phylogenetic trees of the bacterial genera tentatively predicted to encode the biosynthetic gene clusters (BGCs) identified in saliva, subgingival and supragingival plaque samples collected from control and periodontitis subjects.

The identities of the corresponding phyla and predicted BGC products are shown according to the colored key under the diagrams. The radiating bars indicate the number of BGCs associated with each phylum.

Subnetwork BGC cluster alignment

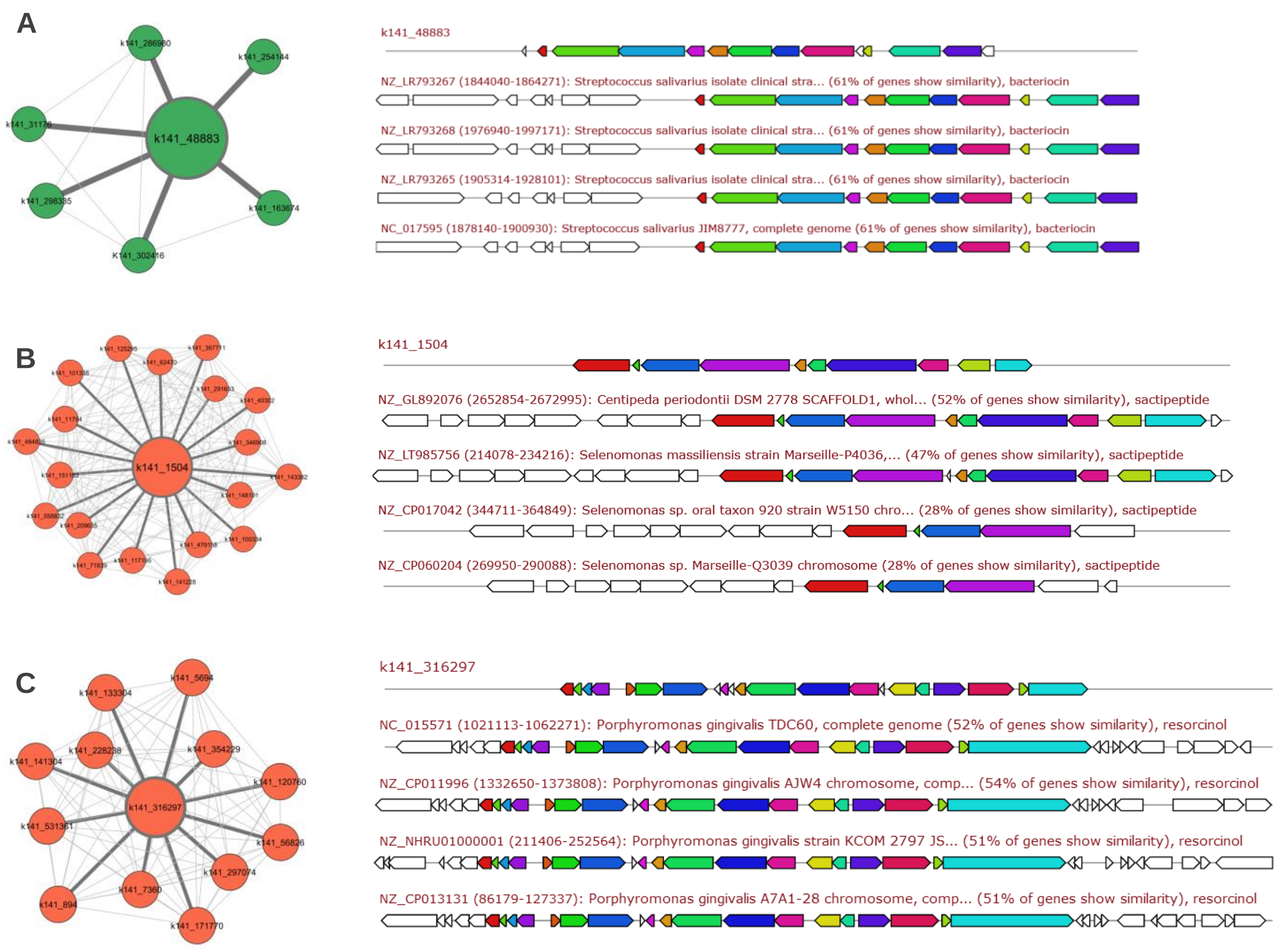

Figure S3. Comparative Network Analysis of Hub BGCs.

This figure illustrates the hub nodes within the biosynthetic gene cluster (BCG) networks derived from control and periodontitis oral microbiome samples. Each hub BGC node has been analyzed against the MiBIG database to infer potential biosynthetic products.

Supplementory Tables:

Table S1. The 44 biosynthetic gene clusters (BGCs) subtypes identified and their mapped superclass (mapped to six super classes)

BGCs	Super class
Arylpolyene	Polyketide
Ripp-Like	RiPP
NRPS	NRP
Terpene	Terpene
NRPS-like	NRP
Resorcinol	Other
RRE-containing	RiPP
Ranthipeptide	RiPP
T3PKS	Polyketide
Lanthipeptide-Class-II	RiPP
Lassopeptide	RiPP
T1PKS	Polyketide
LAP	RiPP
Hserlactone	Other
Thiopeptide	RiPP
Siderophore	Other
Lanthipeptide-Class-III	RiPP
Lanthipeptide-Class-I	RiPP
Transat-PKS-Like	Polyketide
Cyclic-Lactone-Autoinducer	RiPP
Butyrolactone	Other
Transat-PKS	Polyketide
Ladderane	Other
Lanthipeptide-Class-IV	RiPP
Betalactone	Other
hgle-Ks	Polyketide
Ras-Ripp	RiPP
T2PKS	Polyketide
PKS-like	Polyketide
Proteusin	RiPP
NAPAA	NRP
Furan	Polyketide
Phosphonate	Other
Sactipeptide	RiPP
Prodigiosin	Other
Lanthipeptide-Class-V	RiPP
Linaridin	RiPP
Other	Other
Thioamitides	RiPP
Amglyccycl	Saccharide
Ectoine	Other
Nucleoside	Other
Phenazine	Other
Thioamide-NRP	RiPP

Table S2. Periodontal disease diagnosis of the study participants (n = 39) whose sample(s) had purified DNA concentrations ≥ 50 ng/μL (n = 48). (attached as xlsx file)

Table S3. Summary of the 48 shotgun metagenome samples from the 39 study participants (attached as xlsx file)

Table S4. List of the 10,742 bacterial biosynthetic gene clusters (BGCs) identified within the 48 oral samples with purified DNA concentrations ≥ 50 ng/μL (attached as xlsx file)

Table S5. Novel oral biosynthetic gene clusters (BGCs): those with Biosynthetic Genes Super-Linear Clustering Engine (BiG-SLiCE) score > 1500 (n = 207) (attached as xlsx file)

Table S6. Summary of novel BGCs that appear in at least 10 subjects. Enriched column indicates the subject group within which the BGC is enriched.

BGC ID number	Most similar BGC from MIBiG database	Similarity score	Predicted product(s)	Enriched	Host taxon
**k141_317058**	BGC0001238	0.37	biotin	Control	Neisseria
**k141_328908**	BGC0000866	0.26	polyhydroxyalkanoate	Control	Ottowia
**k141_432967**	BGC0002404	0.32	citrinin	Periodontitis	Desulfobulbus
**k141_157704**	BGC0001739	0.24	phosphonoacetic Acid	Periodontitis	Peptostreptococcus
